# Survey of West Nile and Banzi Viruses in Mosquitoes, South Africa, 2011–2018

**DOI:** 10.3201/eid2901.220036

**Published:** 2023-01

**Authors:** Caitlin MacIntyre, Milehna Mara Guarido, Megan Amy Riddin, Todd Johnson, Leo Braack, Maarten Schrama, Erin Gorsich, Antonio Paulo Gouveia Almeida, Marietjie Venter

**Affiliations:** University of Pretoria, Pretoria, South Africa (C. MacIntyre, M.M. Guarido, M.A. Riddin, T. Johnson, L. Braack, M. Venter);; Copperbelt University, Kitwe, Zambia (T. Johnson);; Leiden University, Leiden, the Netherlands (M. Schrama);; University of Warwick, Coventry, United Kingdom (E. Gorsich);; NOVA University of Lisbon, Lisbon, Portugal (A.P.G. Almeida)

**Keywords:** flavivirus, West Nile virus, Banzi virus, *Culex*, Culicidae, disease vectors, vector-borne infections, viruses, zoonoses, South Africa

## Abstract

We collected >40,000 mosquitoes from 5 provinces in South Africa during 2011–2018 and screened for zoonotic flaviviruses. We detected West Nile virus in mosquitoes from conservation and periurban sites and potential new mosquito vectors; Banzi virus was rare. Our results suggest flavivirus transmission risks are increasing in South Africa.

Flaviviruses have been major emerging zoonotic pathogens in Africa within the past decade (*1*). In South Africa, West Nile virus (WNV) is the main flavivirus detected in animals and humans (*2*,*3*). Several other lesser-known flaviviruses were first described in South Africa but are understudied and potentially underreported, including Wesselsbron, Usutu, and Banzi (BANV) viruses (*4*). In South Africa, mosquito surveillance is not routinely performed and studies on flavivirus ecology are outdated (*5*). In this study, we aimed to update flavivirus vector epidemiology in northeastern provinces of South Africa through a large-scale ecologic survey.

## The Study

**Figure 1:**
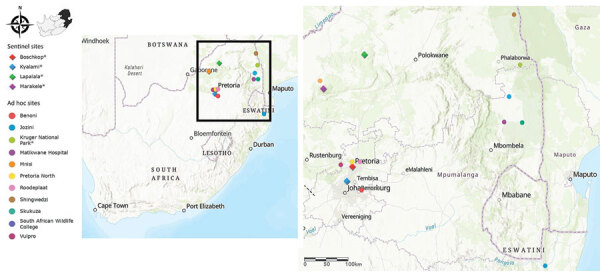
Sentinel and ad hoc mosquito collection sites across the northeastern region of South Africa in survey of West Nile and Banzi viruses in mosquitoes, South Africa, 2011–2018. Collection sites were selected according to recent cases of arboviral disease in humans and animals. Asterisks in the color-coded figure legend indicate sites where flaviviruses were identified in mosquitoes.

We selected 15 sites (4 sentinel sites, 11 ad hoc sites) across 5 provinces in South Africa for mosquito collection according to recent cases of arboviral disease in humans and animals (*2*,*3*) (Figure 1). We established sentinel sites in Boschkop and Kyalami, both located in the Gauteng province (periurban sites), and Lapalala and Marakele, both located in the Limpopo province (conservation sites); collections were performed during 2011–2018 (Table 1). Opportunistic supplementary collections occurred at 10 ad hoc sites during 2015–2018 spanning 3 additional provinces that included urban, periurban, and conservation sites. We performed additional ad hoc collections during March–April 2017 in and around Kruger National Park (KNP) located within Limpopo and Mpumalanga provinces (periurban/conservation sites) (*6*). The methodologies for mosquito collection, identification, pooling, processing, flavivirus screening, and *COX1* gene sequencing have been previously described (*7*). To focus on months from midsummer to autumn in South Africa, when availability of mosquito breeding sites and vectorial capacity increases because of warmer and wetter weather conditions (*8*), we only screened mosquitoes collected during January–June. To submit sequences to GenBank, we generated *NS5* gene fragments >200 bp by using heminested PCR with 0.4 µmol/L each of forward primer (FU1, 5′-TACAACATGGGAAAGAGAGAA-3′) and reverse primer (CFD2, 5′-GTGTCCCAGCCGGCGGTGTCATCAGC-3′) and Platinum Taq DNA Polymerase (Thermo Fisher Scientific, https://www.thermofisher.com). For WNV positive pools, we performed reverse transcription PCR to amplify a 1,525 bp fragment of the WNV envelope protein gene for phylogenetic analysis (Appendix Table). We calculated the mosquito minimum infection rate (MIR) per site by using a standard formula: (number of positive pools/total number of individual mosquitoes tested) × 1000.

**Table 1:** Total number of mosquitoes collected across sentinel and ad hoc sites in survey of West Nile and Banzi viruses in mosquitoes in South Africa during 2011–2018

Site	Site type	Province	Region type	Coordinates	No. mosquitoes collected	No. trapping events	Mean no. mosquitoes/trapping event
Boschkop	Sentinel	Gauteng	Peri-urban	−25.82786, 28.42047	4,790	353	12
Kyalami	Sentinel	Gauteng	Peri-urban	−25.99183, 28.02947	8,736	338	26
Lapalala	Sentinel	Limpopo	Conservation	−23.88458, 28.26953	16,675	568	29
Marakele	Sentinel	Limpopo	Conservation	−24.29364, 27.50325	13,347	487	24
Total nos., sentinel sites					43,548	1,746	23
Benoni	Ad hoc	Gauteng	Peri-urban	−26.10611, 28.36689	1,191	31	38
Jozini	Ad hoc	KwaZulu-Natal	Rural	−27.41258, 32.20647	10,954	47	233
Kruger National Park	Ad hoc	Limpopo, Mpumalanga	Conservation, peri-urban	−25.35384, 31.79936	2,440	30	58
Matikwane	Ad hoc	Mpumalanga	Urban	−24.98545, 31.236342	615	9	68
Mnisi	Ad hoc	Mpumalanga	Rural	−24.48206, 31.38583	5,549	171	32
Pretoria north	Ad hoc	Gauteng	Urban	−25.68663, 28.15895	219	23	12
Roodeplaat	Ad hoc	Gauteng	Peri-urban	−25.62075, 28.37136	298	15	20
Shingwedzi	Ad hoc	Limpopo	Conservation	−23.10819, 31.43628	457	13	35
Skukuza	Ad hoc	Mpumalanga	Conservation	−24.99633, 31.59189	482	15	32
Southern African Wildlife College	Ad hoc	Mpumalanga	Conservation	−24.53886, 31.33369	56	9	15
Vulpro	Ad hoc	Northwest	Peri-urban	−25.7112, 27.95322	490	12	41
Total nos., ad hoc sites					22,751	375	53

We collected a total of 66,299 mosquitoes belonging to 11 genera across 5 provinces in South Africa during 2011–2018 (Table 1; Appendix Figure 1). From those, we divided 40,731 female mosquitoes into 1,471 pools based on morphology, focusing on only 4 genera (*Culex*, *Mansonia*, *Anopheles*, and *Aedes*) and screened for flaviviruses. We detected WNV in 16 (1.09%) and BANV in 2 (0.14%) pools. We did not detect other zoonotic flaviviruses; however, insect-specific flaviviruses were detected and described elsewhere (*7*). WNV outbreaks can be expected once the MIR rises above 1 (*9*). We observed the highest MIRs in Kyalami (periurban site, MIR = 2.53), KNP (periurban/conservation site, MIR = 1.22), and Lapalala (conservation site, MIR = 1.01) (Table 2). Therefore, we identified those areas as higher risk sites for WNV outbreaks in humans and animals. These results correlated with areas where *Culex* spp. mosquitoes were the most abundant and where WNV cases were previously reported in humans and animals in South Africa (*3*,*10*).

**Table 2:** Detection of flaviviruses and their associated potential mosquito vectors in survey of West Nile and Banzi viruses in mosquitoes, South Africa, 2011–2018*

Site	Virus	Positive pool ID	No. mosquitoes†	Morphologic ID‡	Molecular ID§	MIR
Boschkop	WNV	GAU11MP26	2	*Culex pipiens* sensu lato	Not done	0.72
	WNV	GAU17MP72	33	*Cx. univittatus*	*Cx. univittatus*	
Kruger National Park	WNV	KNP17MP714	4	*Cx. simpsoni*	*Cx. simpsoni*	1.23
	WNV	KNP17MP718	36	*Cx. univittatus*	Cx *perexiguus*	
	WNV	KNP17MP720	1	*Cx. bitaeniorhynchus*	*Cx. bitaeniorhynchus*	
Kyalami	WNV	KYA11MP11	14	*Cx. univittatus*	Not done	2.53
	WNV	KYA11MP13	10	*Cx. pipiens s.l.*	Not done	
	WNV	KYA14MP133	10	*Cx. univittatus*	*Cx. univittatus*	
	WNV	KYA14MP134	19	*Cx. univittatus*	*Cx. univittatus*	
	WNV	KYA14MP115	5	*Cx. theileri*	*Cx. theileri*	
Lapalala	WNV	LAP13LP71	44	*Anopheles* spp.	Not done	1.01
	WNV	LAP13LP28	50	*Anopheles* spp.	Not done	
	WNV	LAP13LP22	50	*Aedes* spp.	Not done	
	WNV	LAP14MP394	2	*Cx. univittatus*	*Cx. univittatus*	
Marakele	WNV	MAR13MP77	50	*Cx. poicilipes*	Not done	0.33
	WNV	MAR15MP18	1	*An. gambiae s.l.*	*An. gambiae s.l.*	
Lapalala	BANV	LAP13MP25	49	*Cx.* spp.	*Cx. rubinotus*	0.83
	BANV	LAP13MP26	50	*Cx.* spp.	*Cx. annulioris*	

*BANV, Banzi virus; ID, identification; MIR, minimum infection rate; WNV, West Nile virus. †Number of mosquitoes in each positive pool. ‡Species of mosquito identified by morphologic characteristics. §Species of mosquito identified by sequencing the *COX1* gene.

**Figure 2:**
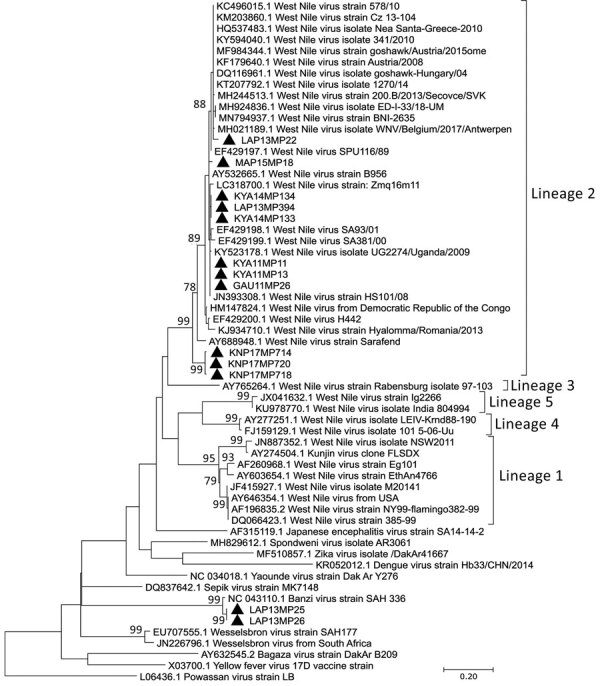
Phylogenetic analysis of flaviviruses using *NS5* gene sequences in survey of West Nile and Banzi viruses in mosquitoes, South Africa, 2011–2018. Maximum likelihood analysis was used to identify flaviviruses found in mosquitoes after partial sequencing of the flavivirus *NS5* gene region (226 nt, Kimura 2-parameter model plus gamma distribution plus proportion of invariable sites). Sequence data were edited by using CLC Main Workbench version 8.0.1 (QIAGEN, https://www.qiagen.com). Reference genomes were downloaded from GenBank. Multiple sequence alignments were created by using MAFFT (https://mafft.cbrc.jp/alignment/server/index.html) with default parameters. Phylogenetic analysis was performed by using MEGA X software (MEGA, https://www.megasoftware.net) with bootstrap support for network groupings calculated from 1,000 replicates. Bootstrap values (>70%) are displayed on branches. GenBank accession numbers for newly sequenced virus strains: OL411950 (KYA11MP13 isolate), OL411951 (GAU11MP26 isolate), OL411952 (KYA14MP133 isolate), OL411953 (KYA14MP134 isolate), OL411954 (LAP14MP394 isolate), OL411955 (MAR15MP18 isolate), OL411956 (LAP13MP22 isolate), OL411957 (KNP17MP714 isolate), OL411958 (KNP17MP720 isolate), OL411959 (KNP17MP718 isolate), OL411960 (KYA11MP11 isolate), OL411961 (LAP13MP25 isolate), and OL411962 (LAP13MP26 isolate). Solid black triangles are new viral sequences that were detected in mosquitoes in this study. Scale bar indicates nucleotide substitutions per site.

Only 11 of 16 WNV-positive pools had partial *NS5* gene sequences of sufficient quality to perform maximum-likelihood analysis; we confirmed all 11 pools were WNV and also confirmed 2 BANV-positive pools (Figure 2; bootstrap value = 100 for both viruses). We observed high nucleotide similarity (94.62%–100.00%) between the identified WNV *NS5* gene sequences and those from previously identified, highly neuroinvasive strains from South Africa isolated from either equines or humans. We successfully amplified the 1,525 nt region of the envelope protein gene for 5 of 16 WNV-positive pools (Appendix Figure 2).

We performed *COX1* gene sequencing for 9 of 16 WNV-positive pools and confirmed morphologic identification of those mosquitoes as *Cx. univittatus* except for 1 pool collected in KNP that was identified by sequencing as *Cx. perexiguus* (Table 2; Appendix Figure 3), a mosquito species not known to be present in South Africa (*11*). This 1 pool might have contained a mix of both species, but Sanger sequencing was unable to distinguish between the 2 species. In addition, the *COX1* reference sequences for *Cx. perexiguus* mosquitoes obtained from online databases may not be accurate because this species has not been identified in South Africa. Recently, *COX1* gene amplification using universal primers followed by next-generation sequencing was shown to distinguish between species in mixed pools (*12*) and might be useful in future studies to resolve this ambiguity. Further studies are necessary to clarify the status of the *Cx. perexiguus* mosquitoes in South Africa. Most of the WNV-positive pools consisted of *Cx. univittatus*, *Cx. pipiens s*.*l*., and *Cx. theileri* mosquitoes, which we collected in high abundance. This result reiterates the importance of these species as WNV vectors in South Africa (*5*). We identified *Cx. simpsoni*, *Cx. bitaeniorhynchus*, *An. gambiae* sensu lato, and *Cx. poicilipes* mosquitoes, none of which have been previously associated with WNV in South Africa, as new potential vectors for WNV by using *COX1* gene sequencing (*4*). Globally, from this list of species, only *Cx. poicilipes* mosquitoes from Senegal were found to be infected with WNV (*13*). Experimental studies have shown that the *Cx. bitaeniorhynchus* mosquito is a likely vector for WNV because this species was able to successfully transmit WNV (*14*). We were unable to genetically characterize the remaining 7 of 16 pools because of insufficient material for DNA extraction; we identified mosquitoes in those pools by morphologic characteristics.

Detection of BANV in mosquito pools in South Africa has not been described since the late 1970s (*15*), and a lack of surveillance raises the question regarding the true incidence of this virus. Only the *Cx. rubinotus* mosquito is recognized as a vector for BANV (*15*). Despite unclear morphologic identification, we identified *Cx. rubinotus* and *Cx. annulioris* mosquitoes in the 2 BANV-positive pools through *COX1* gene sequencing (Table 2; Appendix Figure 3), which should be investigated further to confirm vector status.

## Conclusions

The first limitation of our study is that we did not separate voucher specimens for mosquito species from the pools before homogenization. A voucher specimen is a mosquito species that is preserved and serves as a reference used to document identity. Second, identifications of mosquito species not previously associated with WNV infection are preliminary findings, and further investigation of vector competency is required.

Mosquito surveillance is not routinely performed for arboviruses in South Africa, and most studies were performed 40 years ago (*5*). In this study, 5 provinces were targeted for mosquito surveillance over a 7-year period. These investigations revealed a wide range of new potential vectors that require further investigation. Both WNV and BANV were identified in mosquitoes in periurban and conservation areas at the animal/human interface in South Africa, suggesting increasing circulation potential for those viruses between humans, wildlife, domestic animals, and avian species that are common in those areas. 

Appendix Additional information for survey of West Nile and Banzi viruses in mosquitoes, South Africa, 2011–2018.
