# Supplementary material for: Survey of West Nile and Banzi Viruses in Mosquitoes, South Africa, 2011–2018
Source: Emerg Infect Dis. 2023 Jan;29(1):164–9. doi: 10.3201/eid2901.220036 (PMC9796198; doi:10.3201/eid2901.220036)
Supplement: Appendix — Additional information for survey of West Nile and Banzi viruses in mosquitoes, South Africa, 2011–2018. [file 22-0036-Techapp-s1.pdf]

Article DOI: https://doi.org/10.3201/eid2901.220036

Survey of West Nile and Banzi Viruses in Mosquitoes, South Africa, 2011–2018

Appendix

Appendix Table. PCR primers used to amplify a 1,525-bp fragment of the West Nile virus envelope protein gene for phylogenetic analysis in a survey of West Nile and Banzi viruses in mosquitoes, South Africa, 2011–2018*

Assay	Primer Name	Orientation	Primer sequence, 5′–3′	Fragment size (bp)
RT-PCR, 1st round	WNV1EF	Sense	AAGACAGAATCATGGATACTG	1,853
	WNV1ER	Anti-sense	GCCATTAAGGATGAGCTGAAC	
RT-PCR, 2nd round	WNV2EF	Sense	CATTGGATGGATGCTAGGAAG	1,525
	WNV2ER	Anti-sense	CACATGCAGAAGGAGTCTGCG	

*RT-PCR, reverse transcription PCR.

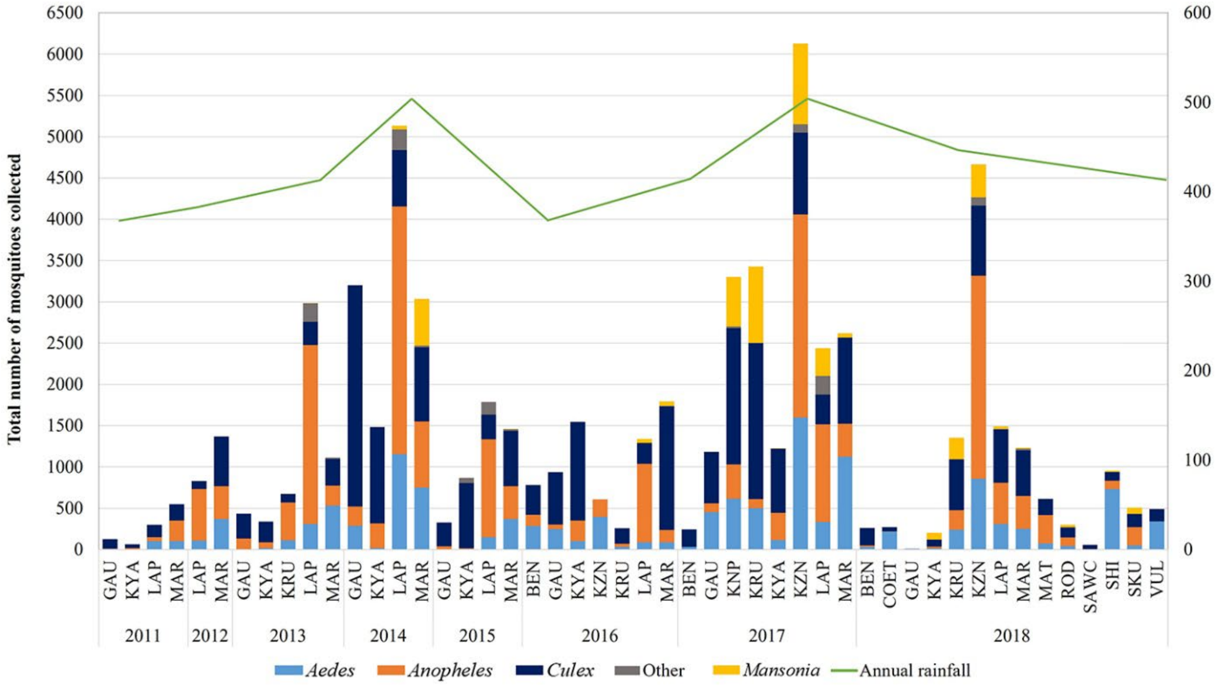

Appendix Figure 1. Distribution of mosquito genera collected annually at sentinel and ad hoc sites in survey of West Nile and Banzi viruses in mosquitoes, South Africa, 2011–2018. The number of mosquitoes collected per site per year is displayed, and the annual average rainfall is for each region is indicated. Annual average rainfall data were obtained from the weather service in South Africa (https://www.weathersa.co.za/home/historicalrain). *Culex* mosquitoes were the most abundantly collected genera. A total of 25,397 (38.31%) *Culex* mosquitoes were collected followed by *Anopheles* (32.52%, n = 21,560), *Aedes* (20.47%, n = 13,573), *Mansonia* (6.79%, n = 4,500), and other genera combined (1.91%, n = 1,269); other genera combined consisted of *Uranotaenia*, *Aedeomyia*, *Ficalbia*, *Coquillettidia*, *Mimomyia*, *Culiseta* and *Eretmapodites*. BEN, Benoni; COET, Pretoria North; GAU, Boschkop; KNP,

Kruger National Park; KRU, Mnisi; KYA, Kyalami; KZN, Jozini; LAP, Lapalala; MAR, Marakele; MAT, Matikwane; ROD, Roodeplaat; SAWC, South African Wildlife College; SHI, Shingwedzi; SKU, Skukuza; VUL, Vulpro.

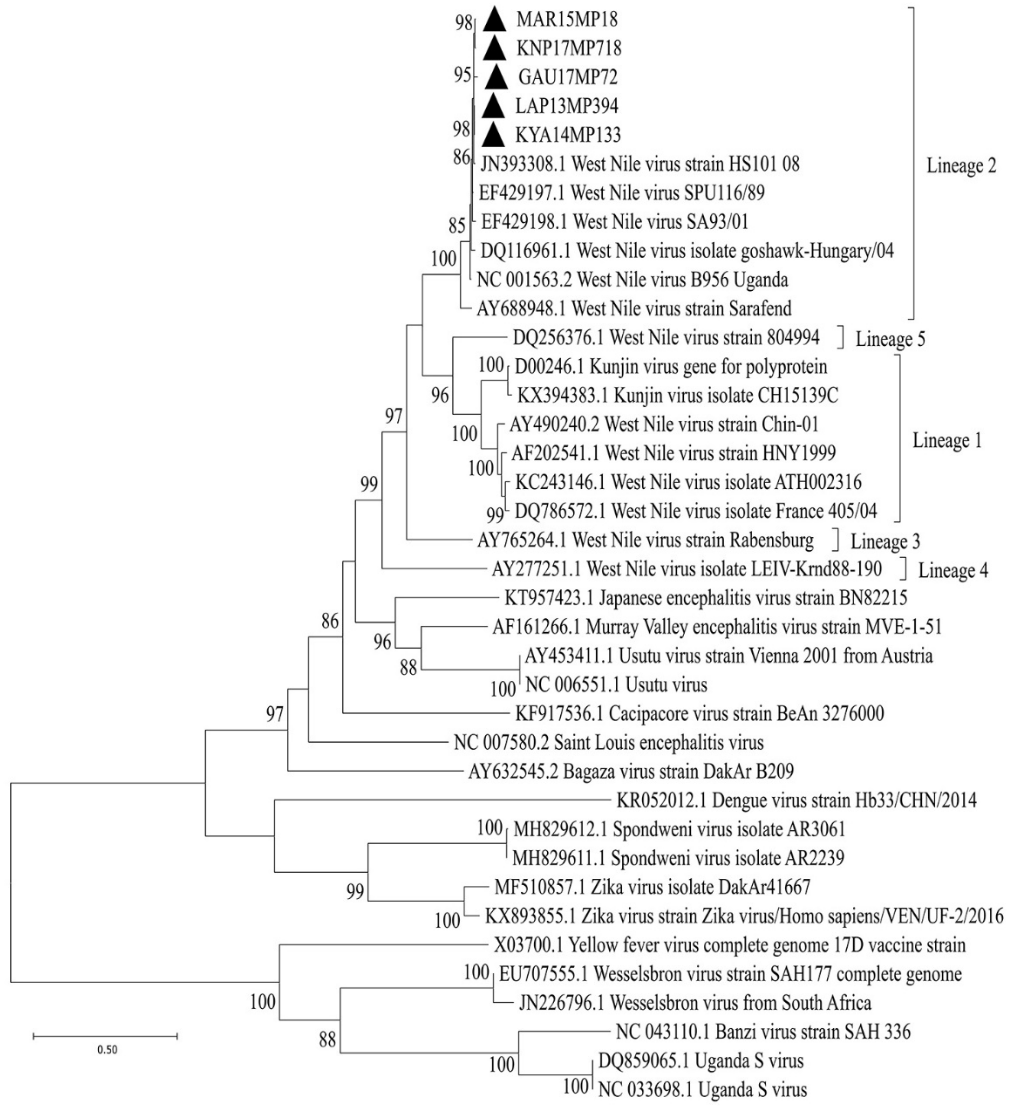

Appendix Figure 2. Phylogenetic analysis of flaviruses using envelope protein gene sequences in survey of West Nile and Banzi viruses in mosquitoes, South Africa, 2011–2018. Maximum likelihood analysis was used to identify flaviviruses found in mosquitoes after partial sequencing of the flavivirus envelope protein gene region (1,440 nt, GTR + G model [general time-reversible model + gamma distribution]). Bootstrap values (>70%) are displayed on the branches. Reference genomes were downloaded from GenBank. GenBank accession numbers for the newly sequenced strains are: OL411963 (MAR15MP18 isolate), OL411964 (KNP17MP718 isolate), OL411965 (LAP13MP394 isolate), OL411966 (KYA14MP134 isolate), and OL411967 (KYA14MP133 isolate). Solid black triangles are new viral sequences detected in mosquitoes in this study. Scale bar indicates nucleotide substitutions per site.

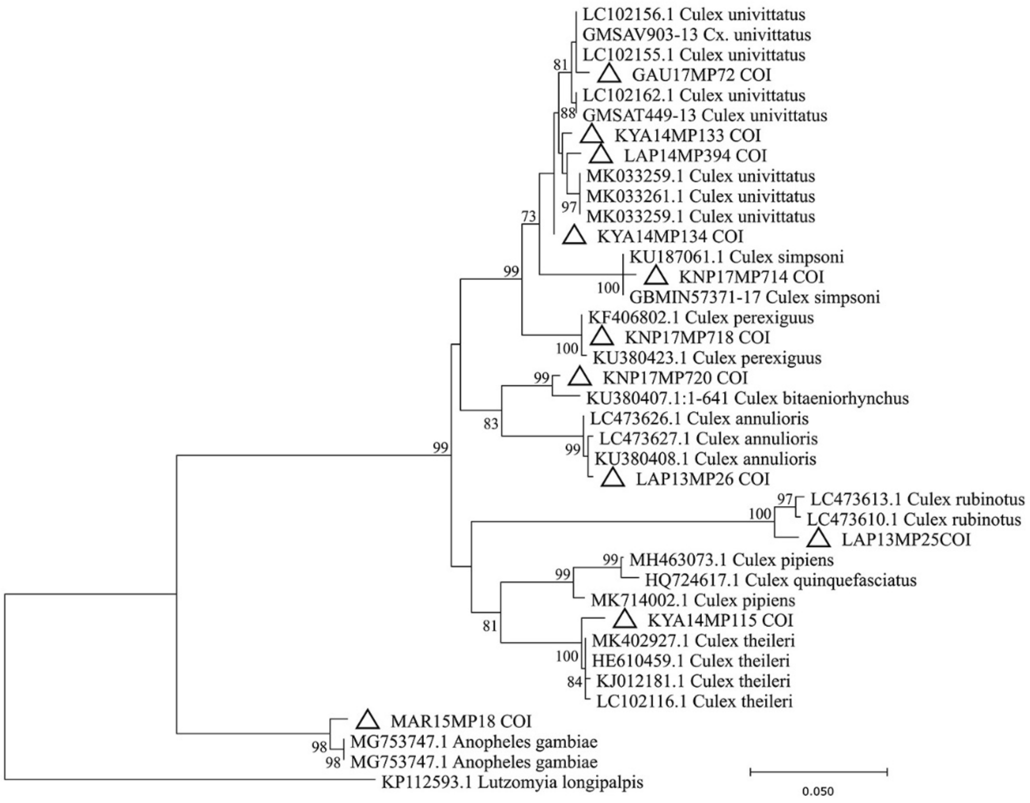

Appendix Figure 3. Phylogenetic analysis of mosquitoes using *COX1* gene sequences in survey of West Nile and Banzi viruses in mosquitoes, South Africa, 2011–2018. Maximum likelihood analysis was used to identify mosquitoes after partial sequencing of the *COX1* gene region (657 nt, GTR + I model [general time-reversible model + gamma distribution]). Reference genomes were downloaded from GenBank or the Barcode of Life Data System (Boldsystems, https://www.boldsystems.org). GenBank accession numbers are: OL457134 (GAU17MP72 isolate), OL457138 (KNP17MP714 isolate), OL457137 (KNP17MP718 isolate), OL457140 (KNP17MP720 isolate), OL457144 (KYA14MP133 isolate), OL457135 (KYA14MP134 isolate), OL457139 (KYA14MP115 isolate), OL457136 (LAP14MP394 isolate), OL457143 (MAR15MP18 isolate), OL457142 (LAP13MP25 isolate), and OL457141 (LAP13MP26 isolate). *COX1* gene sequencing was not performed for GAU11MP26, KYA11MP11, KYA11MP13, LAP13LP71, LAP13LP28, LAP13LP22, or MAR13MP77. Open triangles are *COX1* sequences from mosquitoes collected in this study. Scale bar indicates nucleotide substitutions per site.
